# Development of sample clean up methods for the analysis of *Mycobacterium tuberculosis* methyl mycocerosate biomarkers in sputum extracts by gas chromatography–mass spectrometry

**DOI:** 10.1016/j.jchromb.2015.02.010

**Published:** 2015-04-01

**Authors:** Simona C. Nicoara, Nicholas W. Turner, David E. Minnikin, Oona Y.-C. Lee, Denise M. O'Sullivan, Ruth McNerney, Reggie Mutetwa, Liz E. Corbett, Geraint H. Morgan

**Affiliations:** a Centre for Earth, Planetary, Space and Astronomical Research CEPSAR, The Open University, Milton Keynes, UK; b Life, Health and Chemical Sciences, The Open University, Milton Keynes, UK; c Institute of Microbiology and Infection, University of Birmingham, Edgbaston, Birmingham, UK; d London School of Hygiene & Tropical Medicine, London, UK; e Biomedical Research and Training Institute, Harare, Zimbabwe

**Keywords:** Cholesterol, *Mycobacterium tuberculosis*, Solid phase extraction, Molecularly imprinted polymers, Thermochemolysis, GC–MS

## Abstract

A proof of principle gas chromatography–mass spectrometry method is presented, in combination with clean up assays, aiming to improve the analysis of methyl mycocerosate tuberculosis biomarkers from sputum. Methyl mycocerosates are generated from the transesterification of phthiocerol dimycocerosates (PDIMs), extracted in petroleum ether from sputum of tuberculosis suspect patients. When a high matrix background is present in the sputum extracts, the identification of the chromatographic peaks corresponding to the methyl derivatives of PDIMs analytes may be hindered by the closely eluting methyl ether of cholesterol, usually an abundant matrix constituent frequently present in sputum samples. The purification procedures involving solid phase extraction (SPE) based methods with both commercial Isolute-Florisil cartridges, and purpose designed molecularly imprinted polymeric materials (MIPs), resulted in cleaner chromatograms, while the mycocerosates are still present. The clean-up performed on solutions of PDIMs and cholesterol standards in petroleum ether show that, depending on the solvent mix and on the type of SPE used, the recovery of PDIMs is between 64 and 70%, whilst most of the cholesterol is removed from the system. When applied to petroleum ether extracts from representative sputum samples, the clean-up procedures resulted in recoveries of 36–68% for PDIMs, allowing some superior detection of the target analytes.

## Highlights

• We developed a sample clean-up method to detect tuberculosis from sputum by GC–MS.
• Biomarkers recovered: 64–70% (standards solution), and 36–68% (sputum extracts).
• Cholesterol removed: 93–98% (standards solution) and 62–92% (sputum extracts).
• Less cholesterol in the filtered extracts avoids overloading of the analytical system.
• Analyzing large sample batches will need fewer interruptions for system cleaning.

## 1 Introduction

Tuberculosis (TB) is an infectious disease that is still of increasing concern, with higher incidence in the underdeveloped countries, it represents a world scale problem, given the extensive international mobility of people in the 21st century [1], and its early diagnosis is crucial in reducing and stopping the disease from spreading [2]. The cell envelopes of *Mycobacterium tuberculosis* are rich in unusual lipids, not present in the mammalian tissue. These compounds are therefore considered as proper mycobacterial biomarkers that can enable the disease detection in fluids from infected patients.

Gas chromatographic–mass spectrometric (GC–MS) analysis, following the offline or online derivatization of lipids from the mycobacterial cell membrane, has been proven to be successful for the TB diagnosis by detecting *M. tuberculosis* biomarkers in sputum [3–7]. The 10-methyl octadecanoic acid biomarker, also known as tuberculostearic acid (TBSA), has been frequently analyzed by GC–MS in positive [3–6] and negative chemical ionization (NCI) modes [7], but it is almost ubiquitous among the members of mycolata class, and in other genera such as *Nocardia*. Furthermore, TBSA is dispersed in the cell envelope within a range of plasma membrane phospholipids and lipoglycans, and access to the full content of TBSA requires its prior hydrolytic release by complicated chemical manipulations [3,4,6,7]. Direct analysis of TBSA by GC–MS was reported [5], following thermochemolysis of aliquots from sputum samples, but our experiments based on this procedure [8] resulted in poor sensitivity and specificity, owing to the TBSA ester co-elution with derivatives of other matrix compounds, such as the methyl ester of octadecanoic acid, which is usually present in excessive amounts in sputum.

**Fig. 1:**
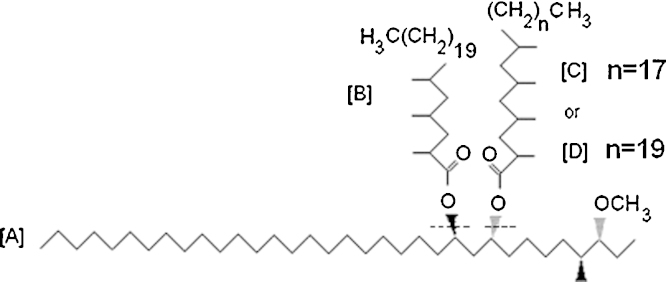
The molecular structure of phthiocerol dimycocerosates (PDIMs), prior to thermochemolysis. The dotted lines show the sites of cleavage during the thermally assisted hydrolysis and methylation process.

There are other classes of lipids which can be immediately accessible for rapid analysis, using simple solvent extraction from culture or sputum sample deposits. Our study involves the more accessible class, the family of phthiocerol dimycocerosates (PDIMs) shown in Fig. 1. They are stable, high molecular weight hydrophobic waxes of around a 90 carbon backbone, which are highly resistant to hydrolysis, and hence will survive the usual procedures used to sterilize infected materials isolated from patients. The mycocerosate components are only present in a limited number of mycobacteria (*Mycobacterium microti*, *Mycobacterium bovis*, *Mycobacterium kansasii*, *Mycobacterium marinum*, *Mycobacterium ulcerans* and *Mycobacterium leprae*) [9–12]. The methyl derivatives of mycocerosates from *M. tuberculosis* give characteristic doublet peaks with dominant C_29_, C_30_, and C_32_ components. In an early study by Larsson, 5-days-old cultures of sputum specimens were shown by GC–MS to have C_32_ mycocerosates [6]. Negative-ion chemical ionization GC–MS methods developed for sensitive detection of mycobacterial mycocerosates [9], has been used to detect mycocerosate biomarkers for ancient tuberculosis in a skeletal collection [13].

**Fig. 2:**
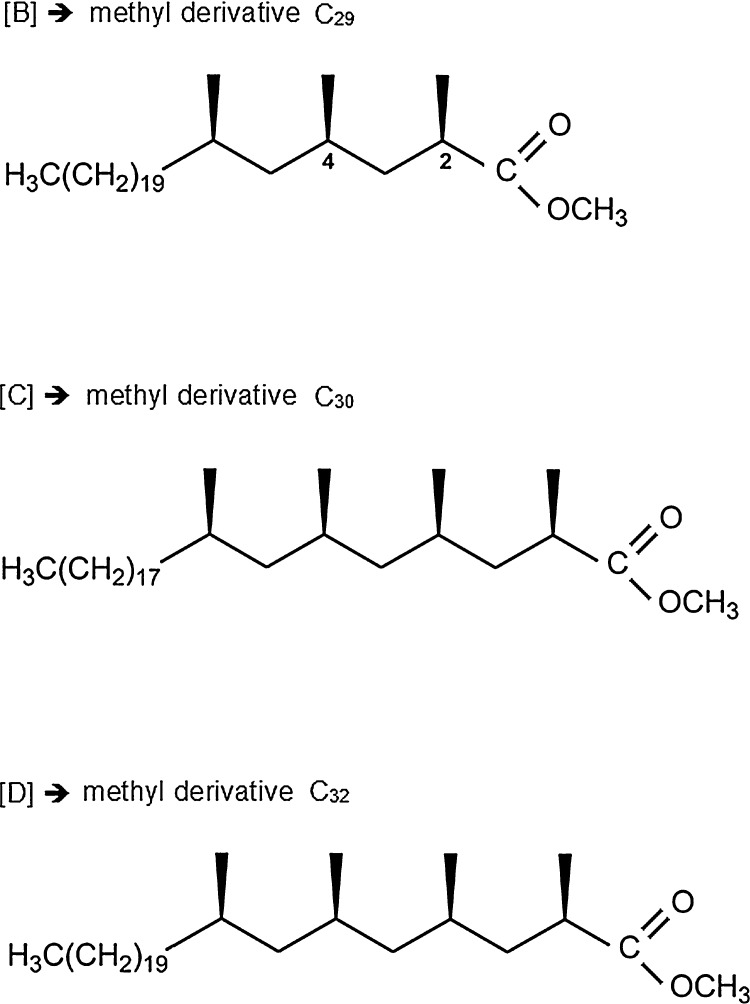
Molecular structures of the target analytes resulted from the transesterification of the PDIM moieties (B)–(D) as shown in Fig. 1, via thermally assisted hydrolysis and methylation in the PTV inlet.

Our work focuses on PDIMs as excellent *M. tuberculosis* biomarkers. Following apolar lipid extraction from sputum, PDIMs were submitted to thermally assisted hydrolysis and methylation (THM) in the programmed temperature vaporizer (PTV) inlet, and the resulting methyl mycocerosates, shown in Fig. 2, were then analyzed by GC–MS. The initial THM-GC–MS method development [14] and its application to a batch of positive and negative real sputum samples [15] were presented in our earlier publications, which focused on the overall method performance without reporting and discussing the particular results obtained in certain sputum sample extracts with a high level of matrix compounds. Blind analysis of the PE extracts of 400+ sputum specimens [15], using our THM-GC–MS method gave 64.9% sensitivity and 76.2% specificity, and it was noted that other components of sputum, such as cholesterol, may hinder the analysis. The presence of matrix peaks in the retention time region of the analytes and a high baseline rendered difficult the assignment of the target doublet peaks, when a low target signal was hidden by abundant background peaks. Derivatized matrix compounds build up in the inlet, which eventually leads to active sites and may compromise subsequent runs. This also impacted on the column life time, causing frequent column overloading and even column blockages, the capillary column needed trimming and/or replacement. Both the PTV inlet and the EIMS ionization source required cleaning on a regular basis, which is not ideal for a routine method. It is therefore desirable to extract PDIMs from the sputum sample, and to filter the extract in order to purify and concentrate them prior to analysis.

In the present work, a proof of principle clean up method was developed, aiming for a maximum recovery of PDIMs and minimum collection of cholesterol from a stock solution of PDIMs and cholesterol standards in PE. The lipid extraction from sputum samples was performed with a combination of apolar petroleum ether (PE) and an immiscible polar solvent (methanol), applying a modified Dobson protocol [10]. Four PE extracts of positive sputum samples were selected that were previously [15] found to have high amounts of cholesterol. These were then passed through different solid phase extraction (SPE) materials, both commercial and molecularly imprinted polymers (MIPs) cartridges. Molecular imprinting is performed by producing a polymeric matrix, containing complementary residues, in the presence of the target molecule [16], in this case cholesterol. After formation, the original template is removed, leaving cavities that are complementary to the shape and chemical profile of the template, only allowing specific recognition and rebinding.

## 2 Experimental

### 2.1 Molecularly imprinted polymer synthesis

Methacrylic acid (MAA), ethyleneglycol dimethacrylate (EGDMA) and azo-N,N′-diisobutyronitrile (AIBN); and all solvents (HPLC grade) were purchased from Sigma Aldrich, (Dorset, UK).

Cholesterol is a favoured template for imprinting studies and has been imprinted by several different researchers [17–19]. A generic MAA/EGDMA methodology was used as proof-of-principle, similar to that of Puoci et al. [17]. For the polymerization procedure, the template (cholesterol), the monomers, cross-linker and free-radical initiator AIBN were dissolved in the porogen chloroform in the ratio 1:4:20 with 125% of mixture to porogen ratio. An amount of 25 mg of template was dissolved into 1.41 mL of chloroform in a 4.5 mL glass vial. To this solution, 21.9 μL of methacrylic acid (monomer) and 1.63 mL of EGDMA (cross-linker) were added and mixed using a vortex. 10 mg of AIBN were added to this solution and mixed via vortex. Once all components had dissolved, the solution was then sparged with nitrogen for 1 min and the vial sealed. Polymerization was carried out at 60 °C for 24 h, in a dry oven. After polymerization, the tubes were then smashed and the monolithic polymer obtained was ground with a mortar and pestle and wet-sieved (methanol) through a series of mesh metal sieve. Particle fractions of 63–38 μm size were collected. Fine particles were removed by repeated sedimentation using acetonitrile. Removal of the imprinted cholesterol from the imprinted particles was undertaken by a Soxhlet extraction with methanol–acetic acid (9:1, v/v) for 48 h. Non-imprinted polymers (NIPs) were synthesized and treated simultaneously under the same conditions without the addition of the template. Empty 3 mL polymeric SPE cartridges were packed with 20 mg of polymer, between two glass fibre frits (20 μm porosity). This general method is adapted from Zulfiqar et al. [20].

### 2.2 Phthiocerol dimycocerosates (PDIMs) standard

The PDIMs standard consists of waxes extracted from freeze-dried *M. tuberculosis* strain C [21], using petroleum ether (PE) from Fischer Scientific (Loughborough, UK) in a biphasic mixture with aqueous methanol [10]. The main component of the PDIMs based on phthiocerol A [22], was purified from the PE extract by preparative thin-layer chromatography [23]. The relative abundances of the C_29_/C_30_ and C_32_ in the standard are not known, hence the quantitative analyses in this study will be reported with respect to the total amount of PDIMs present. Using petroleum ether (PE) (60–80 °C, pesticide residue grade, Distol) from Fisher Scientific (Loughborough, UK) as a solvent, a stock solution was prepared, containing 625 ng mL^−1^ of cholesterol (Acros) from Sigma–Aldrich (Gillingham, UK) and 17.2 ng mL^−1^ of PDIMs standard.

### 2.3 Sputum samples

Sputum specimens were collected from TB patients and suspects at Beatrice Road Infectious Diseases Hospital in Harare, Zimbabwe [24]. Sputum samples were homogenized with glass beads and then split into two: one sample was used for smear microscopy and culture. The second aliquot of sputum was frozen until shipment to London, where they were decontaminated and fully homogenized, using the modified Petroff's method [25]. In brief, 0.5 mL of the sputum samples were homogenized with 0.5 mL of 4% methanolic NaOH, vortexed, incubated at room temperature for 20 min, then neutralized with phosphate buffer, washed in sterile distilled water, and centrifuged, resulting in a 1 mL initial sputum deposit that was heat killed (30 min at 100 °C), then stored at −80 °C prior to the apolar lipids extraction. Defrosted deposits were further concentrated by centrifugation at 14,000 × *g* for 15 min. The supernatant (800 μL) was discarded leaving behind a 0.2 mL deposit that was subsequently submitted to the apolar lipid extraction.

### 2.4 Apolar lipid extraction

Using a modified method from Dobson [10], the apolar lipids were extracted from 0.2 mL of sputum deposits, by adding 1.8 mL methanol and 1 mL petroleum ether (60–80 °C), mixed on a tube rotator for 15 min, then centrifuged at 1200 × *g* for 1 min. The upper PE layer, containing the apolar lipids, was removed and stored at 4 °C prior to the THM-GC–MS analysis.

### 2.5 Sample clean up

Firstly, aliquots of 50 μL of the stock solution of PDIMs and cholesterol standards mix in PE were placed into different commercial SPE cartridges Isolute-Florisil from Biotage UK (Hengoed, UK), then washed in 4 mL of the solvent mixtures (A) or (B) described below. In order to identify the solvent mix with the optimum polarity to wash out the pthiocerol dimycocerosates, while leaving the cholesterol adsorbed on the SPE cartridge, the following solvent combinations were used: (A) dichloromethane and hexane (DCM + H), and (B) heptane with toluene (Hp + T) with the relative concentrations as summarized in Table 1. All the solvents were pesticide residue grade, Distol from Fisher Scientific (Loughborough, UK). Finally, the 4 mL of solvents mix eluted through the SPE cartridges were reconstituted in 50 μL petroleum ether, and were then submitted to the THM-GC–MS analysis. Two positive sputum specimens were identified that had large cholesterol peaks in the original PE extracts. Aliquots of 50 μL from these PE extracts were loaded onto the SPE cartridges, and then the solvent mix that had been selected for the standards clean-up was further tested on the PE sputum extracts selected.

Secondly, 500 μL aliquots of the stock solution of PDIMs and cholesterol standards in PE were washed through molecular imprinted polymers (MIPs) SPE cartridges developed at The Open University, and 50 μL of the first eluent collected (roughly 350 μL) were submitted to the THM-GC–MS analysis. The clean-up method was then tested on two other positive sputum samples, by filtering 500 μL of each PE sputum extract through a MIPs cartridge.

### 2.6 Equipment

An Agilent GC–MS system was used, consisting of a 7890A gas chromatograph equipped with a DB-5 MS capillary column (15 m × 0.25 mm × 0.25 μm) coupled to a 5975C quadrupole mass spectrometer (MS), with the electron impact (EI) ionization source set at 70 eV and 35 μA emission current. The column dimensions in our method were consistent with the end-user operational requirements for its potential use as a diagnostic test in the field. An autosampler PAL-CTC provided with a LINEX liner exchanger was used in conjunction with an Optic3 programmable temperature vaporization (PTV) inlet, to perform the online derivatization of the analytes. Both the autosampler and the inlet were provided by GL-Scientific (former ATAS-GL International) (Eindhoven, The Netherlands).

### 2.7 Thermochemolysis

Fifty microlitre aliquots (i) of the initial stock solution of cholesterol (625 ng mL^−1^) and PDIMs (17.2 ng mL^−1^) in PE solvent; (ii) of the unfiltered PE sputum extracts; and (iii) of each eluent through the SPE cartridges were manually applied to a quartz wool plug, inside separate injector liners. The samples were dried offline, on a hot plate at 60–70 °C for 10 min, and then placed on the Linex autosampler tray. The liners were then loaded into the Optic3 PTV inlet, where the thermally assisted hydrolysis and methylation (THM) of the lipids was performed in helium at 380 °C, following the automated injection of 40 μL methanolic tetramethylammonium hydroxide TMAH. Methanolic TMAH (25%, Acros), purchased from Sigma–Aldrich (Gillingham, UK) was diluted to 12.5% in MeOH (pesticide residue grade, Distol) from Fisher Scientific (Loughborough, UK) and was used for the online derivatization of lipids by THM.

### 2.8 GC–MS analysis

The methyl mycocerosates, resulting from the transesterification of PDIMs via the thermochemolysis process, were separated along the capillary column with a temperature programme of 50 °C (8 min) to 350 °C (1 min) at 30 °C/min, and at a carrier gas flow rate of 1.1 mL of helium per min.

The mass spectrometer was operated to alternatingly collect fragment ions in full scan and selected ion monitoring (SIM) modes. Thus, for each analysis run, the instrument generates two chromatographic traces: the full mass scan, and the SIM trace. The target compounds were detected by collecting the fragment ions *m*/*z* 88, 101 (methyl mycocerosates C_29_/C_30_, and C_32_) in SIM mode, and the fragment ion *m*/*z* 368 extracted from full scan data, for the methyl ether of cholesterol. Considering that the PDIMs are expected to be present at trace levels, their specific ions were collected in SIM mode, thus reducing the instrumental noise by a factor of 10, whereas the much more abundant cholesterol peak was analyzed in the full scan simultaneous mode, thus keeping the number of diagnostic ions in SIM to the minimum required for the biomarker analytes. Quantitation of both the mycocerosic acid methyl esters, and the cholesterol methylated derivative was performed using the integration facility in Agilent ChemStation, by manually measuring the areas of the above named peaks in the SIM chromatogram for *m*/*z* 101, and in full scan for the *m*/*z* 368 fragment ions, respectively.

## 3 Results and discussion

**Fig. 3:**
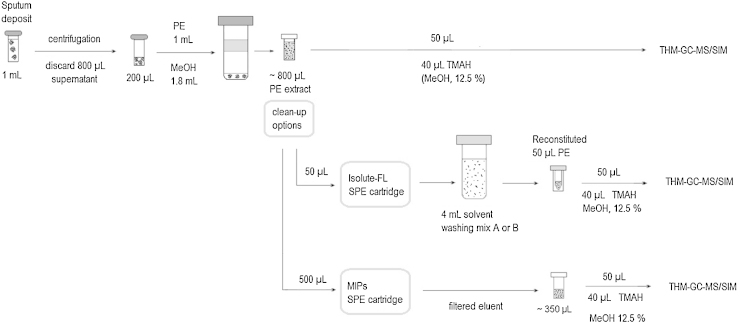
Flow chart from sputum deposits to the THM-GC–MS analysis of the PE extracts. Top: the sample processing and analysis method without clean-up procedure; middle: protocol for the PE extract clean-up through commercial Isolute-FL SPE cartridges; bottom: filtering of the PE extract through developed MIPs materials.

Fig. 3 shows a summary flowchart of the sputum samples clean up procedure as developed and described in Section 2. In essence a multi-step protocol was developed aiming to ensure the cleanest sample possible for injection onto the GC column.

### 3.1 Initial methodology

**Fig. 4:**
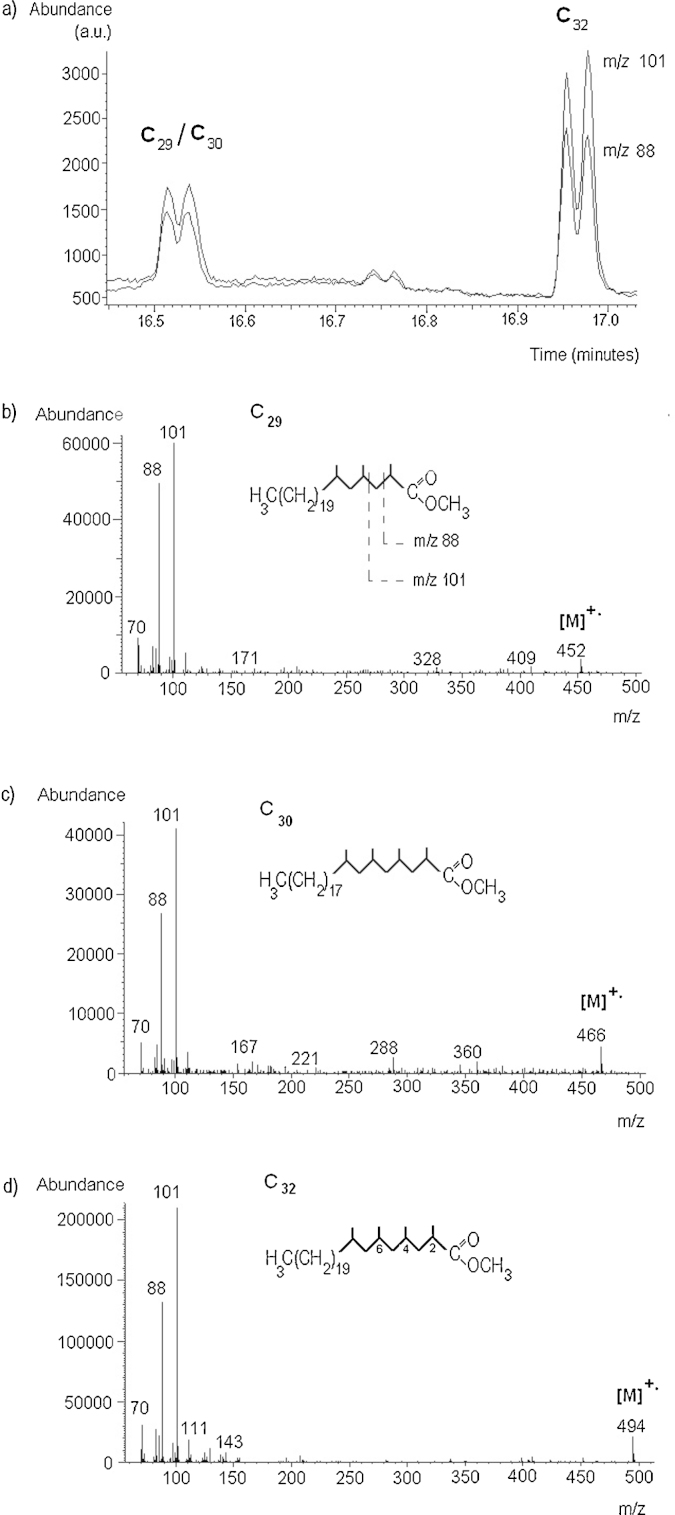
(a) Typical ion chromatograms of the fragment ions at *m*/*z* 101 (top trace) and *m*/*z* 88 (lower trace) obtained in SIM mode for 140 pg total PDIMs submitted to thermochemolysis with 40 μL methanolic TMAH (12.5%). (b)–(d) mass spectra of C_29_, C_30_, and C_32_ methyl mycocerosates, obtained in full scan mode for 4.5 ng total PDIMs under electron impact at 70 eV electron energy.

**Fig. 5:**
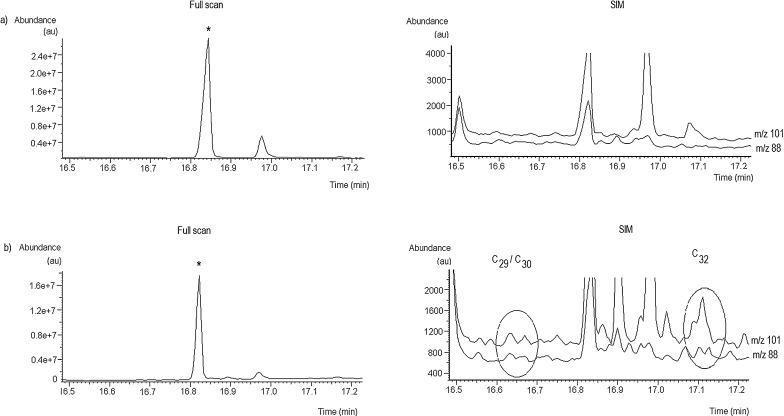
Examples of the GC–MS traces in full scan (left) and SIM (right) for sputum samples with high cholesterol content: (a) a negative sputum (N1); and (b) a positive sputum (P1) (* the cholesterol derivative peak in full scan).

The top line of Fig. 3 shows the current methodologies as described in our prior publications [14,15]. The ion chromatograms for *m*/*z* 101 and *m*/*z* 88 obtained for the methyl derivatives of 140 pg of PDIMs standard are shown in Fig. 4a. The doublet peaks can be observed, which are characteristic to the coeluting diastereoisomers of methyl mycocerosates C_29_ and C_30_, and to the diastereoisomers of C_32_, respectively, as discussed in our earlier publications [14,15]. The mass spectra of C_29_, C_30_, and C_32_ are shown in Fig. 4b–d. Fig. 5 shows some examples of the GC–MS traces in full scan and in SIM, respectively, for sputum samples with high cholesterol content: (a) a negative sputum, and (b) a positive sputum sample extract that saturates the column with both methylated and underivatized cholesterol, identified in the NIST library of mass spectra (98% match).

Given the column overloading by abundant sputum matrix background, a pre-injection clean-up protocol was necessary. Two methods were studied. The first was based around a commercial material (Isolute-FL), a synthetic magnesium silicate that is often used for commercial clean-up protocols. The second was to use a developed MIP material, designed to absorb cholesterol. As discussed above, cholesterol is a common model target for imprinting and a number of papers have developed methodologies. Here we selected an acidic based polymer, similar to the work of Puoci et al. [17], which had demonstrated the ability to selectively bind the target molecule. Given the nature of the PDIM fragments, we expected very little interaction with the polymer, based on the relative charges; however, some non-specific interactions of the PDIMs may also occur with the MIP material.

### 3.2 Isolute-FL (Florisil) extraction

**Fig. 6:**
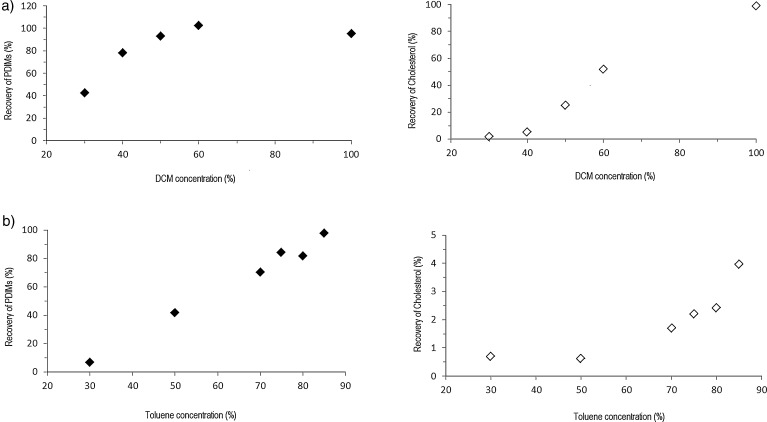
Recovery of PDIMs and of cholesterol from a standards stock solution in PE, following clean up through commercial Isolute-FL SPE cartridges, washed with: (a) washing mix A (dichloromethane:hexane); and (b) washing mix B (toluene:heptane).

Fig. 6a shows the percentage recovery of PDIMs and of cholesterol, using solvent mix A of dicholoromethane (DCM) and hexane (H), with DCM concentration ranging between 0 and 100%. The study aimed to identify the optimum solvent mix polarity relative to that of the SPE packing, to preferentially extract the PDIMs target analytes, while leaving behind most of the cholesterol. Table 1 summarizes the two solvent combinations that we studied, and the various concentrations. In this first attempt, the DCM:hexane mix of solvents at 40% DCM concentration, provided a consistent recovery of 76.4% for PDIMs with only 6.3% for cholesterol. While in temperate and cold climates this solvent mix can be used for clean-up, for the method to be field deployable in countries with high incidence of tuberculosis, hence in tropical countries, we further aimed at replacing the highly volatile DCM (b.p. = 39.9 °C) in a different solvent mix of higher boiling temperatures and also of appropriate polarity.

**Fig. 7:**
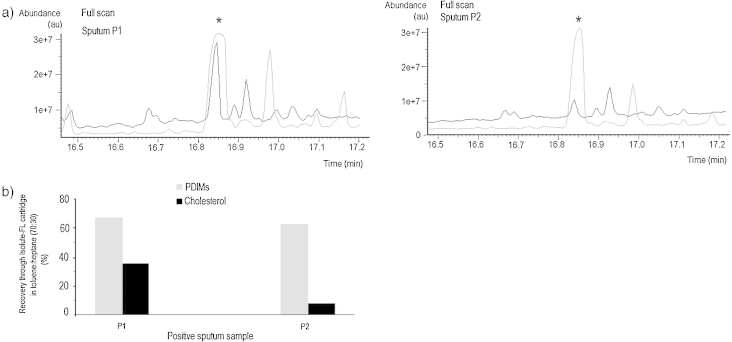
The result of sample clean-up through Isolute-FL commercial SPE cartridges, using a concentration of 70% toluene in a mix with heptane. (a) Comparing the full scan plots of filtered (black), and non-filtered (grey) PE extract of two positive sputum samples with high cholesterol (* = cholesterol derivative peak). (b) Recovery of PDIMs and of cholesterol from two positive sputum samples extracts, washed via Isolute-FL SPE cartridge, with 70% toluene in a mix with heptane.

After testing various less volatile solvents combinations, the preliminary results lead us to select the solvent mix B of heptane (Hp) and toluene (To). Fig. 6b presents the results obtained after sample clean up through Isolute-FL cartridges with combinations of To and Hp. The ideal proportion of these solvents was found to be at 70% toluene, having provided an average recovery of 80.1% for PDIMs and of only 2.05% for cholesterol, from 50 μL of the stock PE solution of standards. When tested on two PE sputum extracts passed through commercial Isolute-FL cartridges, this solvent combination resulted in cleaner chromatograms, as shown in Fig. 7, with recoveries between 38.3 and 68.7% for PDIMs and only 7.8–37.6% for cholesterol. The poorer efficiency of the clean-up with solvent mix B in sputum sample extracts can be due to the matrix complexity and needs to be further investigated. While solvent mix A containing DCM lead to some better results (not shown here) in certain sputum sample extracts, we aimed for a mix of solvents with higher boiling temperatures, that can be used in countries with a high incidence of tuberculosis, and also with warmer climate.

### 3.3 Molecularly imprinted polymer clean-up

**Fig. 8:**
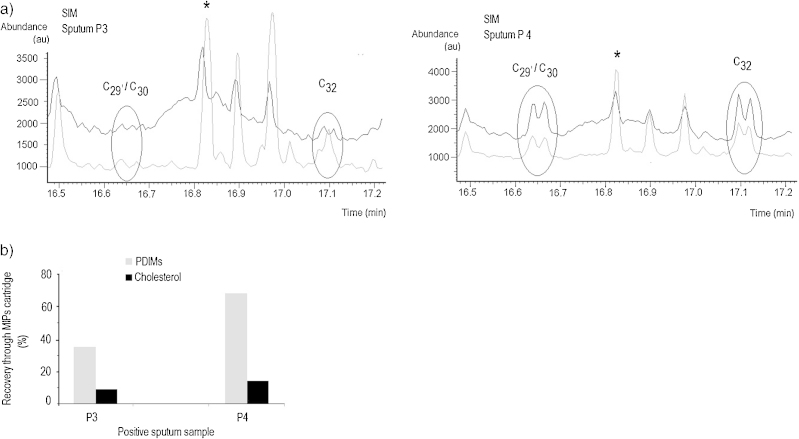
The result of PE sputum sample extracts filtered through MIPs cartridges: (a) comparing the plots of the fragment ion *m*/*z* 101 extracted from SIM scans for filtered (black) and non-filtered (grey) PE extracts of two positive sputum samples (* = cholesterol derivative peak). (b) Recovery of PDIMs and of cholesterol from two positive sputum PE extracts, filtered through MIPs SPE cartridges.

Performing the sample clean-up through MIP cartridges as described in the Experimental section and schematically presented in Fig. 3, is significantly simpler than with the commercial SPE, involving less manipulation of solvents and vials. The THM-GC–MS analysis of 50 μL aliquots of the 350 μL eluent collected from 500 μL standards solution filtered through the MIPs cartridge generated cleaner chromatograms. In the standard stock solution, the clean-up resulted in 64.1–70.6% recovery of PDIMs, and 1.7–6.5% recovery of cholesterol. Fig. 8 shows an example of overlaying the GC–MS traces obtained from two positive sputum sample extracts, by thermochemolysis of 50 μL aliquots, before and after the clean up through the MIPs cartridges. The quantitative results indicate that a significant amount of the cholesterol background has been removed through the filtration of the sputum extracts, with recoveries between 37.5 and 69.7% for PDIMs, and of 8.9–14.5% for cholesterol. Based on the above, we believe that further optimization of MIPs cartridges is needed that could ensure more efficient filtration of PDIMs and removal of cholesterol via a procedure that involves less manipulation of the sample extracts.

These results are very promising for the potential use of SPE clean-up of the PE extracts from real sputum samples, prior to their THM-GC–MS analysis. The chromatograms present reduced matrix peaks, thus improving the conditions to correctly assign the biomarkers signal. One big advantage of using SPE sample clean-up is that this will lead to a lower amount of matrix background introduced in the system, less biologic material deposited on the inlet wall, a reduced risk of column overloading and blockage, and hence enabling a more robust analytical protocol. This allows the analysis of a greater number of sputum samples extracts with a reduced frequency of instrumental interventions for cleaning the inlet and the mass spectrometer ion source, and for column trimming. The present proof of principle assay can further be developed in a systematic study to evaluate the optimum SPE clean-up procedure for an efficient removal of the biological matrix compounds, in order to provide a high sensitivity and selectivity of a more robust and reproducible THM-GC–MS method for the detection of *M. tuberculosis* in sputum samples.

## 4 Conclusions

In order to ease the identification of the target compound biomarkers for tuberculosis in sputum, a proof of principle study was performed of some sample clean-up methods by commercial and MIPs solid phase extraction cartridges, aiming to selectively eliminate the major matrix compounds from the PE sputum extracts.

When used with the proper solvent combinations, the Isolute-Florisil SPE commercial cartridges can remove a high amount of the abundant cholesterol from the sputum extracts, while still recovering more than 70% of the total PDIMs biomarkers present. Though, sample manipulation is more complex and requires relatively longer time than the filtering through MIPs. The MIPs cartridges are relatively easier to use, and the results obtained with standards solution show the potential for selective clean-up of sputum sample extracts.

Both clean-up methods demonstrated the potential to remove the cholesterol from the matrix background present in the PE sputum extracts, without significant loss of the biomarker target analytes. The next step, currently under investigation, is to test this clean-up method to a greater number of sputum samples and apply clinically relevant statistics to the study, to further assess the method performance for the whole sample processing, as promised by the results obtained in the developed methods presented.

## Figures and Tables

**Table 1:** The combinations and concentrations of solvents tested for the optimization of the clean-up assay aiming to maximizing the recovery of PDIMs and the cholesterol removal from a stock solution of standards in PE solvent, through commercial Isolute-Fl (Florisil) SPE cartridges.

Washing mix	Solvent	Polarity index	Boiling temp. (°C)	Solvents concentrations in the washing mix (%)					
A	DCM	3.1	39.9	30	40	50	60	100	
	Hexane	0	68.7	70	60	50	40	–	

B	Toluene	2.4	110.6	30	50	70	75	80	85
	Heptane	0	98.4	70	50	30	25	20	15
